# Establishment of clival chordoma cell line MUG-CC1 and lymphoblastoid cells as a model for potential new treatment strategies

**DOI:** 10.1038/srep24195

**Published:** 2016-04-13

**Authors:** Verena Gellner, Peter Valentin Tomazic, Birgit Lohberger, Katharina Meditz, Ellen Heitzer, Michael Mokry, Wolfgang Koele, Andreas Leithner, Bernadette Liegl-Atzwanger, Beate Rinner

**Affiliations:** 1 Department of Neurosurgery, Medical University of Graz, 8036 Graz, Austria; 2 Department of General Otorhinolaryngology, Head and Neck Surgery, Medical University of Graz, 8036 Graz, Austria; 3 Department of Orthopedic Surgery, Medical University of Graz, 8036 Graz, Austria; 4 Division of Biomedical Research, Medical University of Graz, 8036 Graz, Austria; 5 Institute of Human Genetics, Medical University of Graz, 8036 Graz, Austria; 6 Institute of Pathology, Medical University of Graz, 8036 Graz, Austria

## Abstract

Chordomas are rare malignant tumors that develop from embryonic remnants of the notochord and arise only in the midline from the clivus to the sacrum. Surgery followed by radiotherapy is the standard treatment. As chordomas are resistant to standard chemotherapy, further treatment options are urgently needed. We describe the establishment of a clivus chordoma cell line, MUG-CC1. The cell line is characterized according to its morphology, immunohistochemistry, and growth kinetics. During establishment, cell culture supernatants were collected, and the growth factors HGF, SDF-1, FGF2, and PDGF analyzed using xMAP^®^ technology. A spontaneous lymphoblastoid EBV-positive cell line was also developed and characterized. MUG-CC1 is strongly positive for brachyury, cytokeratin, and S100. The cell line showed gains of the entire chromosomes 7, 8, 12, 13, 16, 18, and 20, and high level gains on chromosomes 1q21–1q24 and 17q21–17q25. During cultivation, there was significant expression of HGF and SDF-1 compared to continuous chordoma cell lines. A new, well-characterized clival chordoma cell line, as well as a non-tumorigenic lymphoblastoid cell line should serve as an *in vitro* model for the development of potential new treatment strategies for patients suffering from this disease.

Chordomas are rare malignant bone tumors that are thought to originate from notochord remnants. They are typically low-grade, locally invasive tumors. They occur at anatomically challenging sites and complete surgical excision is the treatment of choice. Since R0 resection at the base of the skull is seldom possible, surgery should aim for maximum tumor resection with the best possible preservation of neurological function and quality of life^1^. R1 resection should be followed by radiotherapy. In any case, the recurrence rate is high, with skull base chordomas recurring within 29 to 43 months; the 5-year progression-free survival rate is 23–65% with a median overall survival of 6 years^2^. The endoscopic transsphenoidal route and the 4-hand technique provide far better conditions than open surgery for gentle but efficient resection of skull base tumors and preservation of tissue architecture^3^,^4^.

The new personalized treatment options call for *in vitro* models but clivus chordoma cell cultures are difficult to breed and no cell lines are commercially available. To close this gap, we used a full endoscopic technique and created suitable culture conditions, enabling us to establish the new and unique MUG-CC1clivus chordoma cell line.

To investigate the microenvironment of MUG-CC1 right from the outset, we determined growth factors from the supernatant. Cells in the tumor’s microenvironment, for example cancer-associated fibroblasts, directly stimulate tumor cell proliferation by contributing various growth factors, hormones, and cytokines. Classical mitogens are HGF, SDF-1, FGF2, and PDGF^5^. Hepatocyte growth factor (HGF) is produced by mesenchymal cells; the receptor for HGF is cMET, a transmembrane tyorosinase receptor. The HGF/cMet signaling system has been associated with tumorigenesis, disease progression, and invasiveness in many human carcinomas^6^,^7^,^8^ and sarcomas^9^,^10^,^11^,^12^. Stromal cell-derived factor-1 (SDF-1) was originally identified as a bone marrow SDF from stromal cells including immune cells, pericytes, endothelial cells, inflammatory cells, stroma cells, and fibroblasts^13^. The basic fibroblast growth factor (FGF2) could potently stimulate tumor cell proliferation via FGFR signaling^14^,^15^, and FGF2 is also a potent stimulator for the platelet-derived growth factor (PDGF)^16^.

In chordoma research, there is a particular lack of non-tumorigenic human cell lines, so lymphoblastoid cells serve to provide a continuous source of basic biomolecules and a system to carry out various experiments. We were able to establish an actively proliferating B-lymphoblastoid cell line, MUG-CC1-LCL, which is now available for studies on non-tumorigenic cells for genetic and long-term genotype-phenotype correlation studies.

We present a well-characterized clivus chordoma cell line, MUG-CC1, as well as the spontaneously immortalized B-cell line MUG-CC1-LCL. That the cell system could be established was greatly due to a cell-sparing, fully endoscopic surgical technique. The described *in vitro* model will help to further understand the pathogenesis and tumor biology of skull base chordomas and to facilitate development of future treatment strategies.

## Results

### Establishment of cell lines

**Figure 1:**
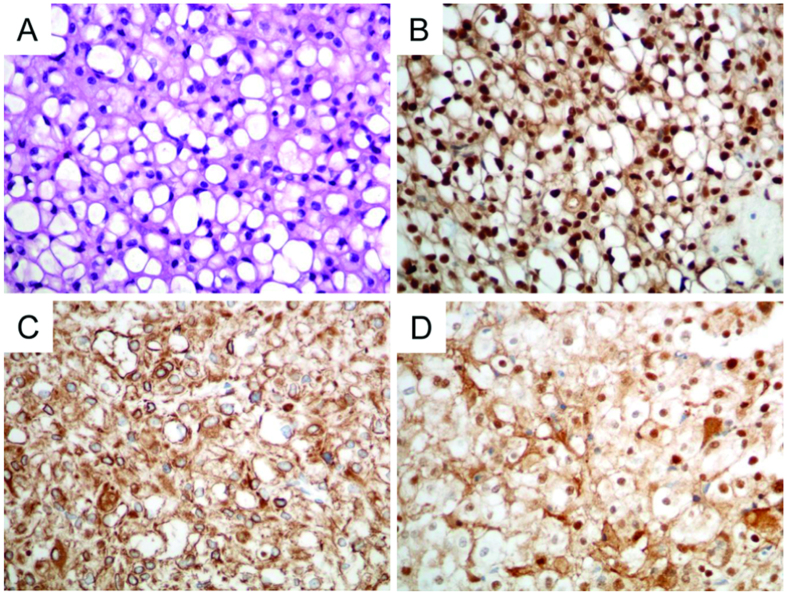
Histology and IHC of the patient´s primary clival tumor used for the establishment of MUG-CC1. (**A**) Hematoxylin and eosin staining. Chordomas show a prominent myxoid matrix and embedded cords and strands of tumor cells with eosinophilic cytoplasm as well as physaliphorous cells. (**B**) Specific nuclear expression of brachyury; co-expression of (**C**) cytokeratin and (**D**) S-100 protein as IHC profile of chordomas.

The diagnosis of classic chordoma was obtained by H&E morphology (Fig. 1A). Immunohistochemical (IHC) stains revealed nuclear positivity for brachyury (Fig. 1B) as well as positive staining for cytokeratin (Fig. 1C), and S100 (Fig. 1D). After mechanical disaggregation, bits of the tumor were cultured in appropriate medium and a mixture of cells developed after only one day. The cells were always kept very tight, and after 14 days single tumor cells were visible. After reaching 90% confluence cells were split by Accutase® or trypsin. After approximately five months, suspension cells were also determined in the cell culture flasks in addition to the tumor cells. These were isolated from the adherent cells and both were cultured and characterized separately. The suspension phenomenon was seen in several MUG-CC1 culture flasks.

### Characterization of clivus chordoma tumor cell line MUG-CC1

**Figure 2:**
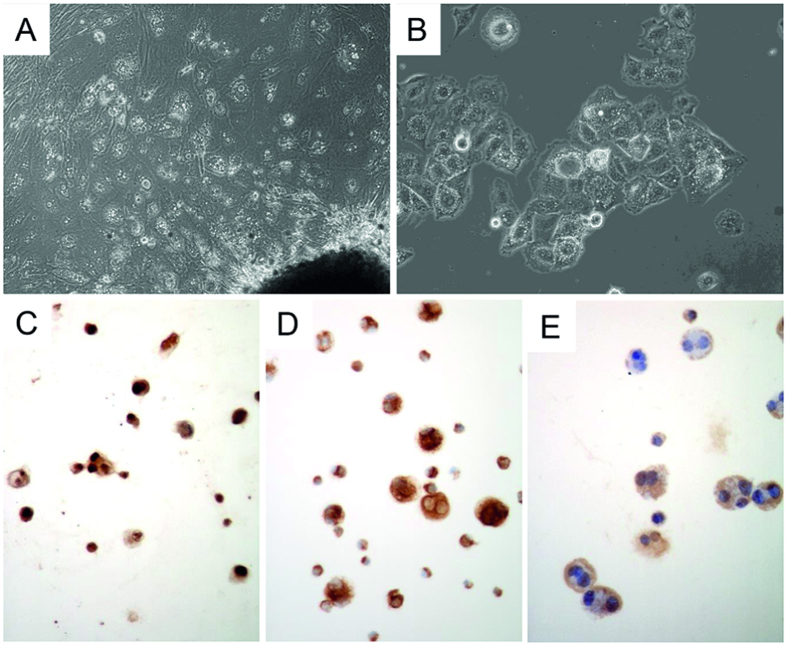
Morphological features of MUG-CC1. (**A**) Outgrowing of primary clival chordoma cells. (**B**) Typical physaliphorous appearance of chordoma cells. (**C**) Cultured chordoma cells show a specific nuclear brachyury expression, (**D**) strong cytokeratin expression, and (**E**) strong vimentin expression by IHC.

After a one-year cultivation period, the MUG-CC1 cell line was established and the chordoma cells were characterized phenotypically. MUG-CC1 cells had a typical physaliphorous appearance and vaculated cytoplasm (Fig. 2A,B). The cultured MUG-CC1 cells showed typical nuclear expression of brachyury (Fig. 2C), with co-expression of cytokeratin (Fig. 2D) and vimentin (Fig. 2E). Morphology, IHC staining profile, and molecular studies were identical to the parental tumor tissue (Fig. 1). MUG-CC1 was maintained in culture for at least 24 months with passage 19. The doubling time of the cell population was determined by the xCELLigence system and was about 10 days at outset; later the continuous cell line MUG-CC1 showed a doubling time of 109.16 ± 18.79 hours.

Five months after cultivation, the cells’ growth behavior was seen to change. During that period, the supernatant was taken and growth factor (HGF, SDF-1, PDGF, and FGF2) analyses were done using xMAP^®^ technology. The very dense cell lawn started to draw back and tumor cells were visible, whereas suspension cells grew in large organizations in the supernatant (Suppl. Fig. 1). Tumor cells displaced fibroblasts and two cell populations - one adherent and one in suspension - originated. We were able to establish and characterize both types of cells: the MUG-CC1 clivus chordoma cells and the MUG-CC1-LCL lymphoblastoid cell line in the suspension. Immunohistochemistry demonstrated that these suspension cells, designated as MUG-CC1-LCL, did not express brachyury (data not shown).

### Characterization of lymphoblastoid cell line

**Figure 3:**
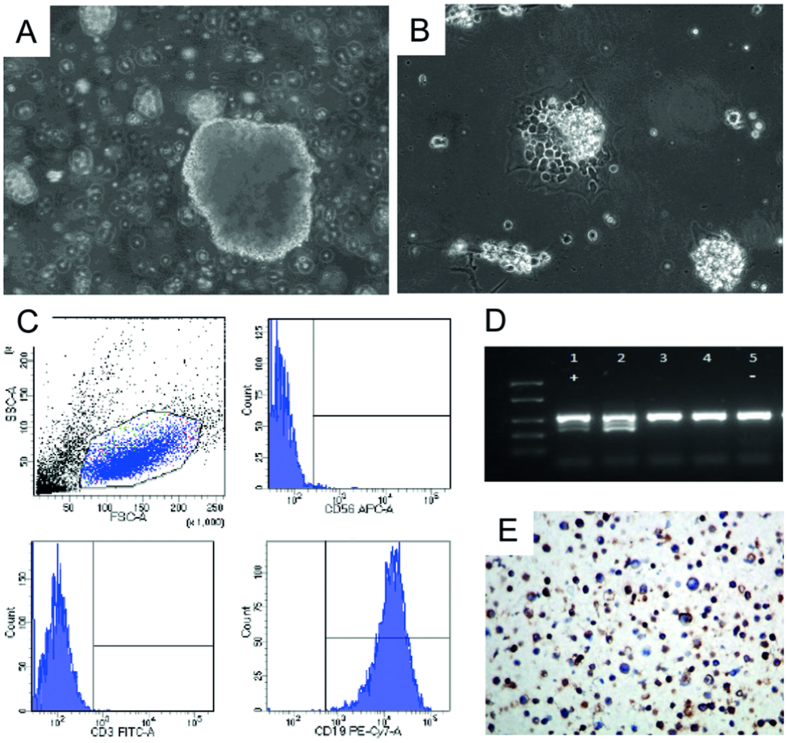
Establishment of the lymphoblastoid cell line MUG-CC1-LCL. (**A**) Morphological analysis; (**B**) single cells and cell clumps are observed; (**C**) Flow cytometry presents forward and side scatter blot, CD56 negative, CD3 negative, and CD19 positive cells. (**D**) EBV detection by PCR. Lane 1: pos. control cells, Lane 2: MUG-CC1-LCL, Lane 3: MUG-CC1 cell line, Lane 4: MUG-CC1 primary tissue, and Lane 5: neg. control (**E**) IHC of MUG-CC1-LCL showed significant positivity of EBV.

The MUG-CC1-LCL grew in a typical rosette morphology in suspension clusters (Fig. 3A)^17^; along with single cells (Fig. 3B). Flow cytometry analyses were positive for B cell marker (CD19), while markers for T cells (CD3) and natural killer cells (CD56) were absent (Fig. 3C). DNA ploidy of MUG-CC1-LCL was assessed spiked with mononuclear cells (MNC) from healthy donor cells and showed a diploid DNA profile (data not shown). Furthermore, copy number profiling showed a balanced profile, suggesting a non-tumorigenic cell line. Strong positivity for the EB2 (BMLf1) gene of EBV (TC 70 and TC 72) was detected in MUG-CC1-LCL, whereas MUG-CC1 and primary tumor showed no EBV expression by RT-PCR (Fig. 3D). Data were confirmed by IHC positivity of EBV (Fig. 3E). All mycoplasma tests were negative.

### Growth factors

**Figure 4:**
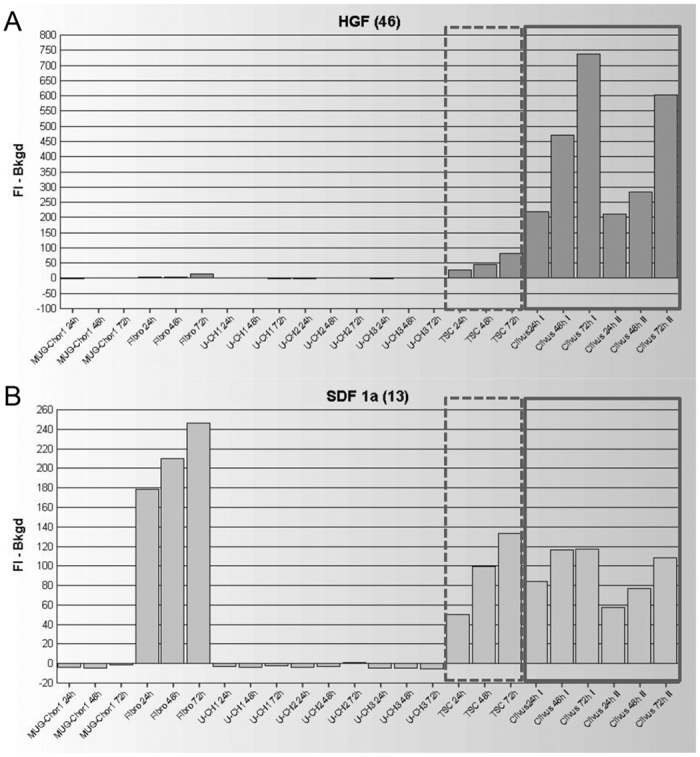
Growth factors detection by xMAP^®^ technology. Supernatant of chordoma cell lines (MUG-Chor1, U-CH1, U-CH2, U-CH3), human skin fibroblasts (fibro), and tumor surrounding cells (TSC) compared with the clival chordoma MUG-CC1 after 24, 48, and 72 h. Clivus I represents the supernatant after these time points, clivus II the repeated measures after a medium change. (**A**) Significant expression of HGF in TSC (dashed box) and MUG-CC1 (unbroken box) compared to the other tested cells and (**B**) SDF-1 in fibros, TSC (dashed box) and MUG-CC1 (unbroken box) were detected.

Within five months, before the cells were separated in two populations, the supernatant was collected to identify important growth factors for establishing a cell culture and to optimize growth media for establishment of further cell lines. Because connective tissue cells were visible during cultivation, we measured HGF, FGF2, SDF-1, and PDGF compared to stable chordoma cell lines (MUG-Chor1^18^ and U-CH1-3^19^) and healthy normal skin fibroblasts (fibro), as well as tumor-surrounding cancer cells (TSC). We detected a time-dependent (24–72 hours) significant increase of HGF in TSC, especially as MUG-CC1 was becoming established, compared to stable chordoma cell lines and fibroblasts (Fig. 4A). There was a significant increase of SDF-1 in primary MUG-CC1, TSC, and in fibroblasts compared to the other cells as well (Fig. 4B). The fluorescent intensity (FI) minus background was presented in all measurements. The FI signals of FGF2 (0–26 FI) and PDGF (0–3.5 FI) were barely detectable or were part of the FI background signal (under the detection limit) and therefore had no effect on the cell cultures (Suppl. Fig. 2).

### Copy number profiling

**Figure 5:**
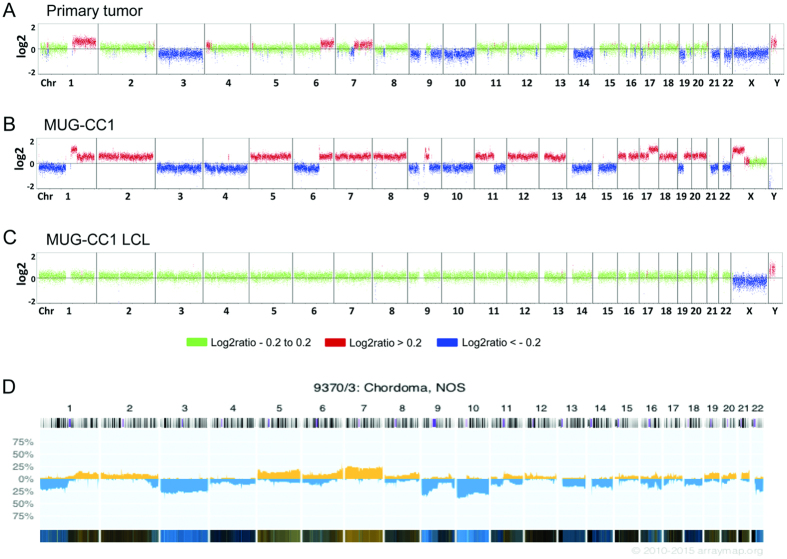
Copy number profiles of the primary clival tumor (**A**) and the newly established cell lines MUG-CC1 (**B**) and MUG-CC1-LCL (**C**). Depicted are log2 ratio plots of the genome. Regions with log2 ratios > 0.2 that indicate gains of chromosomal material are shown in red and those with log2 ratios < −0.2 that indicate loss of chromosomal material are shown in blue. Balanced genomic regions are depicted in green. In contrast to the primary clival tumor and MUG-CC1, which showed a variety of CNA frequently occurring in chordomas (**D)**, publically available copy number data for s from Progenetix database, MUG-CC1-LCL showed a balanced copy number profile suggesting a non-malignant origin.

In order to characterize the different cell lines at the genetic level, we analyzed copy numbers using low-coverage whole genome sequencing. Genome wide copy numbers were inferred from the counts observed across the genome. The primary tumor showed a variety of copy number aberrations (CNAs); i.e. gains of chromosome 1q and 6q as well as losses of chromosomes 3, 9p, 10, 14, and 21 (Fig. 5A) that were highly concordant with available copy number data for chordomas from the public Progenetix database (Fig. 5D). Most of the CNAs from the primary tumor were also seen in MUG-CC1 although at a higher amplitude. This was not an unexpected result since IHC analyses revealed a fraction of approximately 20% non-neoplastic cells in the tumor sample compared to the highly enriched tumor cells in the cell line. The cell line showed some additional changes that were not present in the primary tumor, e.g., gains of the entire chromosomes 7, 8, 12, 13, 16, 18 and 20, (Fig. 5A,B) and high level gains on chromosomes 1q21–1q24 and 17q21–17q25. Interestingly, we observed complete loss of the Y-chromosome in combination with a gain of the X-chromosome including a high level gain of Xp11-Xp22. Most likely these putative *de novo* CNAs represent cell line artifacts that arose during the passaging of cell cultures or represent potential somatic mosaicisms that were cloned during the establishment of the cell culture. Chordomas are characterized by high heterogeneity and during culturing certain clones might prevail while others fail to survive. In addition, unlike a pure tumor population in the cell line, the primary tumor sample is contaminated with normal cells, so that some changes might be underrepresented in the tumor sample. This is further supported by the fact that CNA identified in the cell line show a striking similarity to CNA found in a set of primary tumor samples (n = 51) from the Public Progenetix database. Copy number analysis of the MUG-CC1-LCL cell line revealed a balanced copy number profile, suggesting a non-neoplastic origin of these cells.

### Cell line identification

The frozen primary parental tumor tissue, MUG-CC1 and the MUG-CC1-LCL showed the same tandem repeat (STR) profile at the markers D3S1358, TH01, D18S51, D5S818, D13S317, D7S820, D16S539, CSF1PO, Penta D, vWA, D8S1179, TPOX, and FGY. The cell line showed a loss at Penta E allele 13, D21S11 allele 30, and Amelogenin Y. The STR analysis clearly demonstrated that MUG-CC1 and MUG-CC1-LCL originated from the same patient material (Table 1). Comparisons of the SNP patterns of the mitochondrial DNA confirmed the common origin of all samples.

## Discussion

In recent years, there has been a major surge in chordoma research, thanks to *in vitro* models developed by several groups, though clival chordoma cells are especially difficult to maintain in long-term cultures^20^. Chordoma cell lines are useful for studying the molecular oncogenesis of these chemo-resistant tumors and will likely support the development of new treatment options for patients suffering from this rare disease.

Since chordomas arise from remnants of the embryonic notochord^21^, there are no non-tumorigenic notochordal cells for comparative investigations and it is unlikely that such cell lines will be available in near future. We present for the first time a non-tumorigenic, spontaneously established lymphoblastoid cell line originating from the same chordoma patient.

A variety of well-characterized sacral chordoma cell lines are available for chordoma research^18^,^19^,^22^; however, only one non-commercially available clival cell line (UM-Chor1) has been approved by the chordoma foundation (http://www.chordomafoundation.org/reagents-data/cell-line-repository/um-chor1). The need for additional clival cell lines is underscored by the fact that the most common tumor locations are in the clivus (32%) followed by the sacrum or coccyx (29%). Other less prevalent localizations of chordomas are the cervical (neck), thoracic (upper back), and lumbar (lower-back) vertebrae of the spine^2^.

One possible explanation for this could lie in the role of aggressive telomerase in chordoma. It might be that permanent cell lines could only be obtained from telomerase-expressing chordomas, underlining their aggressive growth behaviour^20^. Cell lines can only be grown from healthy cells, but clival chordoma surgery can destroy or damage cells, so that they are difficult to outgrow. The four-handed transnasal transsphenoidal purely endoscopic technique was the basis for the establishment of MUG-CC1 and offered optimal conditions for the spontaneous formation of the lymphoblastoid cell line, and the spontaneously grown MUG-CC1-LCL provides material for a variety of assays. There are many publications describing the use of lymphoblastoid cell lines as a source of biomolecules like DNA, RNA, and proteins^23^, while DNA isolated from lymphoblastoid cell lines has been used for mutation analysis^24^,^25^ and in addition has provided a valuable, cost effective *in vitro* model for genotypic and phenotypic assays^17^. MUG-CC1-LCL was strongly positive for EBV, which confirmed the immortalization of the lymphoblastoid cells. In cancer biology, an optimal microenvironment is known to play an important role in the growth, proliferation and survival of cancer cells^26^. On the one hand, growth factors and cytokines are important components of the microenvironment, especially of cancer-associated fibroblasts that, on the other hand, can be cultured over long periods. A population of oral mucosal fibroblast doubles in 80–110 days and skin fibroblasts do so in 40–65 days^27^, meaning that fibroblasts can easily be cultured for up to five months and directly affect the growth of tumor cell lines.

Pronounced expression of HGF and cMET was found in skull base chordomas. HGF/cMET signaling systems might be correlated with proliferation, invasion, advanced stage of disease, and furthermore might play a role in tumor progression in a wide spectrum of human malignancies^28^. Since chordoma cells grow slowly, they are very difficult to culture, so that opportunities to study the supernatant of visible growing clival chordoma cells together with tumor stroma cells are rare. Among other growth factors, we investigated HGF expression at the beginning of culture where TSC as well as chordoma cells were present. Proto-oncogen c-MET is located at chromosome 7q31, whereas the gain of chromosome 7q is one of the most common chromosome aberrations in chordoma^19^,^29^,^30^. This indicates that cMET expression via chromosome 7 amplification may play a role in the early stage of skull base chordoma. MUG-CC1 indicates a gain of chromosome 7, and together with the high expression of HGF at culture start, emphasizes the importance of the cMET/HGF signaling system and should be examined further. In a recent publication, the increase in tumor cell proliferation was mediated by SDF-1, secreted by TSC^31^. We detected SDF-1 overexpression in human fibroblasts as well as in TSC, whereas FGF2 and PDGF were under the detection limit.

In conclusion, we present the new, well characterized MUG-CC1 clival chordoma cell line as well as the spontaneously immortalized MUG-CC1-LCL B-cell line. The well characterized system of two cell lines from one patient can be used for further research on clival chordoma treatment.

## Methods

### Surgical approach and harvesting of tissue

The interdisciplinary skull base unit of the Medical University of Graz is an international reference center for endoscopic skull base surgery and has contributed substantially to the development of four-handed transnasal transsphenoidal purely endoscopic approaches. The experience of our center is based on 14 operated clival chordomas as of 2014 via a purely endoscopic transsphenoidal approach with a four-handed technique. The focus of the current study lies in careful tissue preservation and procurement of sufficient cell material as a prerequisite for optimal cell culturing. After appropriate access to the clival region and tumor exposure, preliminary tumor debulking was performed. The specific advantage of this surgical approach lies in the direct visualization of the lesion and the opportunity to obtain non-contaminated (by blood, mucus, mucosa, etc.) and structurally intact tumor tissue from the vital tumor mass.

### Patient history

The primary cells for the establishment of the cell line were obtained from a 72-year-old male patient who was referred to the Department of Neurosurgery because of a right-sided abducent nerve palsy with double vision, vertigo, and cephalea. Magnetic resonance imaging of the head showed a 2.9 × 2.8 × 2.7 cm retro-, supra-, and parasellar tumor infiltrating the right cavernous sinus. Endocrinological evaluation showed a hypogonadotropic hypogonadism. The right-sided abducent nerve palsy was neuro-ophthalmologically confirmed in addition to the newly diagnosed trochlear nerve palsy. There was no evidence of a visual field deficit. An endoscopic transsphenoidal resection of the suspected clival chordoma was indicated and preoperative informed consent was obtained to harvest tumor material for cell culture. Histological examination confirmed a clival chordoma, classic subtype. Despite the cavernous sinus infiltration and cranial nerve involvement, a partial resection was possible. There were no intraoperative complications; a temporary postoperative Schwartz Bartter syndrome responded to appropriate treatment. The abducent nerve palsy improved and there was no additional postoperative cranial nerve deficit. The patient received proton beam therapy and was stable throughout a follow-up period of 22 months. All experiments were conducted and approved in accordance with the guidelines of ethics committee of the Medical University of Graz (votum #18–192ex06/07; valid until 17.04.2016).

### Cell culture

The tumor tissue was obtained immediately after surgery, followed by mechanical disaggregation of the tumor tissue into approximately 1–2 mm^3^ pieces. Cells were cultured in Iscove/RPMI 4:1 (Life Technologies, Carlsbad, CA) containing 10% fetal bovine serum (FBS; Biochrom AG, Berlin, Germany), 1% insulin, transferrin, sodium selenite (ITS; Life Technologies), 2 mM glutamine, and 1% penicillin/streptomycin (Pen/Strep; Life Technologies). The chordoma cells were grown at pH 7.4 to 80% confluence, and detached from the flasks with Accutase® (Sigma, Vienna, Austria). The human skin fibroblasts (fibro) were isolated from the MUG-Chor 1^18^ patient´s upper arm and cultured in DMEM (Life technologies), 10% FBS, and 2 mM glutamine. Tumor surrounding cells (TSC) were isolated from a chordoma patient, cultured in DMEM, 10% FBS, and 2 mM glutamine. MUG-CC1-LCL was cultured in DMEM, 10% FBS, and 2 mM glutamine. The continuous cell lines MUG-Chor1 and U-CH1^19^ were cultured in Iscove/RPMI 4:1 containing 10% FBS, 1% ITS, 2 mM glutamine and were used as control. All cells were incubated at 37 °C in a humidified atmosphere of 5% CO_2_ and were periodically checked for mycoplasma by PCR.

### xCELLigence system

The xCELLigence DP device from OLS (Omni Life Science GmbH & Co. KG, Bremen Germany) was used to monitor cell proliferation in real time. MUG-CC1 cells, 1 × 10^4^ and 2 × 10^4^ were seeded on electronic microtiter plates (E-Plate™; OLS) and measured for 171 h with the xCELLigence system following the user’s manual. Cell density measurements were performed in duplicate with programed signal detection every 20 min. Data were acquired and analyzed with RTCA software (version 1.2, Roche Diagnostics).

### Growth factors xMAP® technology

Concentrations of HGF, SDF-1, FGF2 and PDGF were simultaneously quantified in cell culture supernatant using the ProcartaPlex™ immunoassay (eBioscience, San Diego, CA). Cytokine concentrations were determined using analyte specific capture beads coated with target-specific capture antibodies according to the manufacturer’s specifications. The analytes were detected by biotinylated analyte-specific antibodies. Following binding of the fluorescent detection label, the reporter fluorescent signal was measured with the Bio-Plex 200 multiplex suspension array system and detected with Bio-Plex 5.0 Software (Bio-Rad, Hercules, CA). The sensitivity for the respective cytokines was: HGF: 36,000 pg/ml, SDF-1: 70,000 pg/ml, FGF2: 20,000 pg/ml, PDGF: 30,000 pg/ml. Complete growth medium was used as background control.

### Immunohistochemistry (IHC)

IHC studies using the streptavidin-biotin peroxidase complex method were carried out with the established antibodies against S-100 (Dako, Glostrup, Denmark), cytokeratin (Dako), vimentin (Linaris, Wertheim, Germany), EMA (Dako), and brachyury (Santa Cruz, Santa Cruz, CA), as a recently detected specific marker for chordomas. Appropriate positive and negative controls were included. To confirm the EBV positivity of the LCL cell line by IHC, the antibody monoclonal mouse Anti-Epstein-Barr Virus (Dako-EBV, LMP, CS1-4, M897) was used with a dilution of 1/50 and detected with the K5001 assay (Dako) and the ready-to-use Chromogen AEC (Dako). Protease Type XXIV (Sigam, Munich, Germany) 0.01% in PBS was used for 10 minutes as pretreatment.

### Copy number profiling

Genome-wide copy number aberrations (CNA) were established using low-coverage whole genome sequencing. Shotgun libraries were prepared using the TruSeq DNA LT Sample Preparation Kit (Illumina, San Diego, CA) with slight modifications to the manufacturer’s protocol. Briefly, 380 ng, 144 ng, and 360 ng input DNA from the primary tissue, MUG-CC1, and MUG-CC1-LCL, respectively, were fragmented in 130 μl using the Covaris System (Covaris, Woburn, MA). After concentrating the volume to 50 μl end repair, A-tailing and adapter ligation were performed following the manufacturer’s instructions. For selective amplification of the library fragments that had adapter molecules on both ends, we used 15 PCR cycles for the more highly concentrated samples, i.e. primary tumor and MUG-CC1-LCL and 25 PCR cycles for the MUG-CC1. Libraries were quality checked on an Agilent Bioanalzyer using a DNA 7500 Chip (Agilent Technologies, Santa Clara, CA) and quantified using qPCR with a commercially available PhiX library (Illumina) as a standard. Libraries were pooled equimolarily and sequenced on an Illumina MiSeq in a 150 bp single read run. On the completion of the run, data were base called, demultiplexed on the instrument (provided as Illumina FASTQ 1.8 files, Phred +33 encoding), and FASTQ format files in Illumina 1.8 format were used for downstream analysis. Copy number analysis was performed as previously described. Briefly, low-coverage whole-genome sequencing reads were mapped to the pseudo-autosomal-region (PAR)-masked genome and reads in different windows were counted and normalized by the total number of reads. We further normalized read counts according to the GC content using LOWESS statistics. To avoid position effects we normalized the sequencing data with GC-normalized read counts of a set of 30 non-malignant control samples^32^. We subsequently generated segments of similar copy-number values by applying circular binary segmentation (CBS) and Gain and Loss Analysis of DNA (GLAD). For each segment, we calculated z-scores by comparing GC-corrected read counts for samples and controls.

### Cell line identification Power Plex® 16 system

Frozen tumor tissue was dissected into small pieces and re-suspended in 180 μl ATL buffer from Qiagen (Vienna, Austria). The cell pellet (3.5 × 10^5^) from MUG-Chor1 (p20) was re-suspended in 200 μl PBS, 20 μl proteinase K. Afterwards, 200 μl AL buffer from Qiagen was added. DNA preparations were carried out using the QIAamp DNA Mini kit (Qiagen) according to the manufacturer’s instructions and 1 μl DNA was amplified using the Power Plex 16 System (Promega, Vienna, Austria). The product was mixed with Hi-Di formamide (Applied Biosystems Inc., Foster City, CA) and Internal Lane Standard (ILS600), denatured, and fractionated on an ABI 3730 genetic analyzer. Resulting data were processed and evaluated with the ABI Genemapper 4.0.

### Flow cytometry

For flow cytometry analysis, 2 × 10^6^ cells/ml were re-suspended in a final volume of 150  μl PBS. The commercial monoclonal antibodies 5 μl CD3 FITC, 5 μl CD19 PE-CY7, and 5 μl CD 56 APC, (BD Bioscience, San Jose, CA) were applied for characterization. Background staining for antibodies was performed in negative cell lines and with matched fluorochrome-conjugated isotype controls. Flow cytometry analysis was performed on a FACS LSR II System (BD Bioscience) and data were acquired using FACSDiva software (BD Bioscience). The day-to-day consistency of measurements was checked by Rainbow Beads (BD Bioscience). Viable cells were gated on forward scatter (FSC) and side scatter (SSC) to exclude debris and cell aggregates.

### EBV detection by PCR

PCR was applied for the detection of specific DNA sequences of the human pathogenic virus EBV. DNA from MUG-CC1, MUG-CC1-LCL, and RAJI was isolated applying the QIAamp DNA Mini Kit (Qiagen). Specific oligonucleotide pairs from the EB2 (BMLf1) gene of EBV (TC 70 and TC 72) were used with the appropriate annealing temperatures in the PCR reaction. The PCR was done with the Hot Star Taq Plus Mastermix Kit (Qiagen). After the initial cycle, 35 cycles were run at 95 °C for 30 seconds, template specific annealing temperature for 30 seconds and 72 °C for 1 min plus 2 sec extension for each cycle. The amplified products were identified by agarose gel electrophoresis and visualized by SYBR Gold. Internal control was kindly provided by the DSMZ (Leibniz, Germany). Genomic DNA of the RAJI cell line was used as positive control DNA.

## Additional Information

**How to cite this article**: Gellner, V. *et al.* Establishment of clival chordoma cell line MUG-CC1 and lymphoblastoid cells as a model for potential new treatment strategies. *Sci. Rep.* **6**, 24195; doi: 10.1038/srep24195 (2016).

## Supplementary Material

Supplementary Information

## Figures and Tables

**Table 1:** STR analysis of MUG-CC1 and MUG-CC1-LCL.

	Tumor Tissue	MUG-CC1	MUG-CC1 LCL
D3S1358	16,18	16	16,18
TH01	9.3	9.3	9.3
D21S11	28,30	28	28,30
D18S51	12	12	12
Penta E	10,13	10	10,13
D5S818	11,12	11,12	11,12
D13S317	12,13	12,13	12,13
D7S820	8,11	8,11	8,11
D16S539	9,14	9,14	9,14
CSF1PO	11	11	11
Penta D	10,13	13	10,13
AMEL	XY	X	XY
vWA	16,18	16,18	16,18
D8S1179	13,15	13,15	13,15
TPOX	8,11	8,11	8,11
FGA	23	23	23
