# Supplementary material for: Establishment of clival chordoma cell line MUG-CC1 and lymphoblastoid cells as a model for potential new treatment strategies
Source: Sci Rep. 2016 Apr 13;6:24195. doi: 10.1038/srep24195 (PMC4829844; doi:10.1038/srep24195)
Supplement: Supplementary Information [file srep24195-s1.pdf]

Establishment of clival chordoma cell line MUG-CC1 and lymphoblastoid cells as a model for potential new treatment strategies

Verena Gellner*[1], Peter Valentin Tomazic*[2], Birgit Lohberger[3], Katharina Meditz[4], Ellen Heitzer[5], Michael Mokry[1], Wolfgang Koele[1], Andreas Leithner[3], Bernadette Liegl-Atzwanger[6], Beate Rinner[4]

[1] Department for Neurosurgery, Medical University of Graz, 8036 Graz, Austria

[2] Department of General Otorhinolaryngology, Head and Neck Surgery, Medical University of Graz, 8036 Graz, Austria

[3] Department Orthopedic Surgery, Medical University of Graz, 8036 Graz, Austria

[4] Division of Biomedical Research, Medical University of Graz, 8010 Graz, Austria

[5] Institute of Human Genetics, Medical University of Graz, 8010 Graz, Austria

[6] Institute of Pathology, Medical University of Graz, 8036 Graz, Austria

**Verena Gellner and Peter Valentin Tomazic contributed equally to this article*

corresponding author:

Beate Rinner, Ass.Prof. PhD, MSc.

Medical University of Graz, Division of Biomedical Research,

Stiftingtalstrasse 24, A-8010 Graz, Austria,

phone: +43-316-385-73524

fax: +43-316-385-73009

email: beate.rinner@medunigraz.at

Results

Five months after cultivation, a change in the growth behavior of the cells was observed. During this period, the supernatant was taken and growth factor (HGF, SDF-1, PDGF, and FGF2) analyses were done by xMAP® technology. The very dense cell lawn started to draw back and tumor cells were visible, whereas suspension cells grew in large organizations in the supernatant (Suppl Fig.1). Tumor cells displaced fibroblasts and two cell populations - one adherent and one in suspension - originated. We were able to establish and characterize both types of cells: the clivus chordoma cells MUG-CC1 and the suspension lymphoblastoid cell line MUG-CC1-LCL.

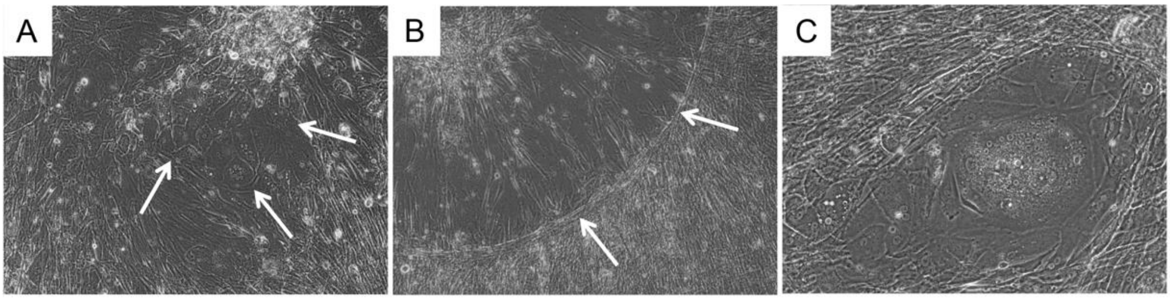

Supp. Figure S1: A) The outgrowth of chordoma cells was indicated by arrows. B,C) Arround the tumor cell cluster TSC were clearly delineated (arrows).

Within five months, before the cells were separated in two populations, the supernatant of cells was collected. This was done to investigate important growth factors during cell culture establishment and to optimize growth media for further cell line establishments. Because connective tissue cells were visible during cultivation, we measured HGF, FGF2, SDF-1, and PDGF compared to stable chordoma cell lines (MUG-Chor1[18] and U-CH1-3[19]), healthy normal skin fibroblasts (fibro), as well as tumor-surrounding cancer cells (TSC). The

fluorescent intensity (FI) minus background was presented in all measurements. The FI signals of FGF2 (0-26 FI) and PDGF (0-3.5 FI) were barely detectable or were part of the FI background signal (under the detection limit) and therefore had no effect on the cell cultures (Suppl Fig.2).

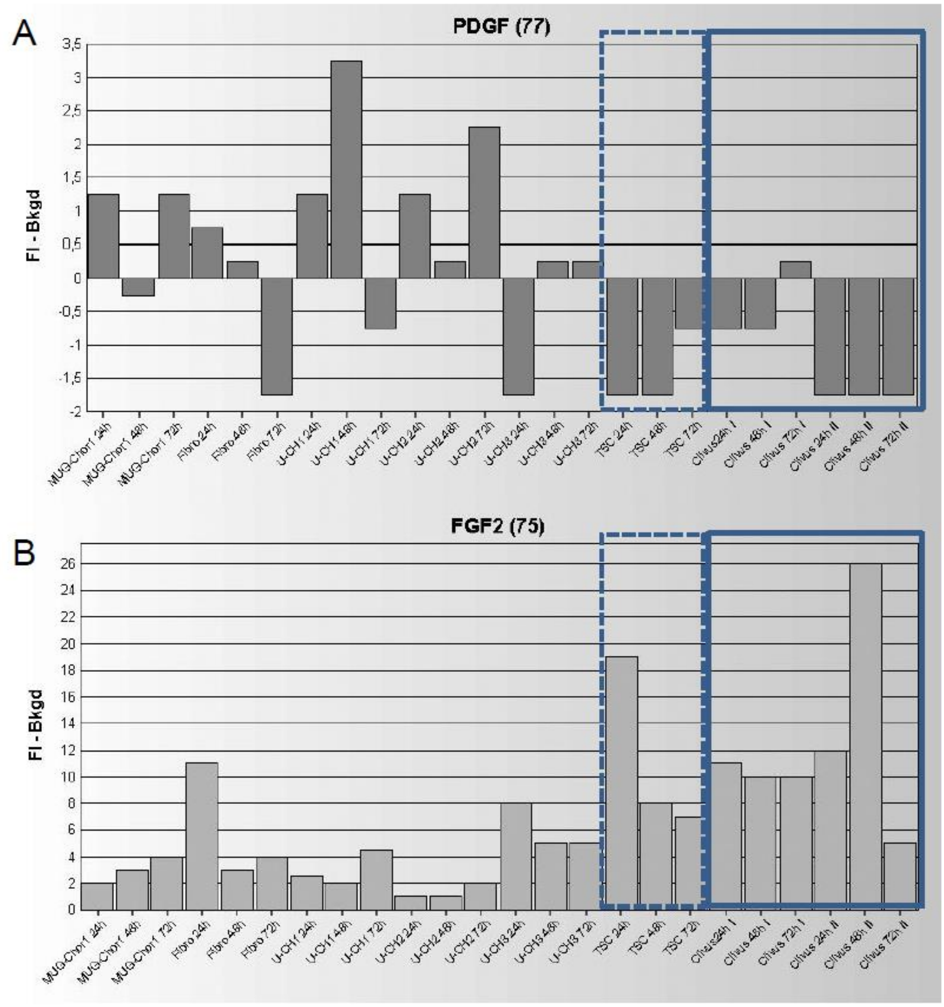

Suppl. Figure S2. Growth factors detection by xMAP® technology. Supernatant of chordoma cell lines (MUG-Chor1, U-CH1, U-CH2, U-CH3), human skin fibroblasts (fibro), and tumor surrounding cells (TSC) compared with the clival chordoma MUG-CC1 after 24, 48, and 72 h. Clivus I represents the supernatant after these time points, clivus II the repeated measures after a medium change. A) Expression of PDGF in TSC (dashed box) and MUG-CC1 (unbroken box) compared to the other tested cells and B) FGF2 in fibros, TSC (dashed box) and MUG-CC1 (unbroken box) were detected.
